# Knockdown of aberrantly expressed nuclear localized decorin attenuates tumour angiogenesis related mediators in oral cancer progression model *in vitro*

**DOI:** 10.1186/1758-3284-4-11

**Published:** 2012-04-16

**Authors:** Nyla Dil, Abhijit G Banerjee

**Affiliations:** 1 Departments of Medical Microbiology and Infectious Diseases, Winnipeg, MB, Canada; 2 Immunology, Winnipeg, Canada; 3 Oral Biology, University of Manitoba, Health Sciences Centre, Winnipeg, MB, Canada; 4 Center for Genomic Bio-Medicine and Res. Inst, Durg, Chhattisgarh, India

**Keywords:** Oral cancer, Angiogenesis, MMP9, CXCR, Decorin, ANG1, VEGF, IL-8, Functional genomics

## Abstract

**Background:**

Oral cancer accounts for roughly 3% of cancer cases in the world with about 350,000 newly reported cases annually and a 5-year survival rate of only 50%. Majority of oral cancers are squamous cell carcinomas that originate in the oral mucosal epithelial linings. We have previously shown that in human malignant squamous cells carcinoma (SCC-25) as well as in dysplastic oral keratinocytes (DOK), a small leucine-rich multifunctional proteoglycan decorin is aberrantly expressed and localized in the nucleus where it interacts with nuclear epidermal growth factor receptor (EGFR). Post-transcriptional silencing of nuclear decorin significantly reduced IL-8 and IL8-dependent migration and invasion in these dysplastic and malignant oral epithelia. The objective of this study was to further examine the effects of nuclear decorin silencing on angiogenesis and angiogenesis related mediators in this oral cancer progression cell line model.

**Methods:**

We have used multiplex PCR, western blotting, and *in vitro* endothelial tube formation assay to study angiogenesis and related pathways in nuclear decorin silenced (stable knockdown) DOK and SCC-25 cells.

**Results:**

Nuclear decorin knockdown resulted in significant down regulation of IL-8 expression, however IL-10, and TGF-β expression was not affected in either DOK or SCC25 cells as measured by multiplex RT PCR. IL-8 receptor CXCR 1 and 2 expression was slightly lower in nuclear decorin silenced cells indicating a contributing mechanism in previously shown reduced IL-8 mediated migration and invasion phenotype in these cells. IL-8 is known to induce Matrix metalloproteinase 9 (MMP9) which not only plays a role in tumour migration and invasion but also induces angiogenic switch. We found MMP9 to be significantly reduced in nuclear decorin silenced dysplastic and malignant oral epithelia. Other potent angiogenic mediators, VEGF_189_ and ANG-1 were either significantly reduced or completely abrogated in these cells. Angiogenesis as measured by endothelial tube-like formations of HUVEC cells was reduced by almost 50 percent when HUVECs were incubated in the presence of conditioned medium form nuclear decorin silenced dysplastic and malignant cell lines as compared to respective controls.

**Conclusions:**

Together these results indicate that aberrantly expressed nuclear localized decorin strongly influences angiogenic potential of dysplastic and malignant oral epithelial cells.

## Background

Squamous cell carcinoma originating in the oral mucosal linings, account for more than 90% of oral cavity cancers [1,2]. The incidence of oral cancer, although not well documented, is on the rise adding to the existing burden due to widespread low survival and high recurrence rates. Oral cancer makes up approximately 3% of all cancer cases [3] and is the 8th most common cancer in the world among men and the 14th most common among women [4]. The incidence rate of oral cancer is highest in Pakistan, France, India, and Brazil [4] and it is responsible for roughly 125,000 deaths annually of which an estimated 95,000 occur in developing countries [3].

A critical step in the pathology of tumor progression is angiogenesis; the generation of new blood vessels from preexisting vessels, without which tumours cant grow beyond couple of mm in size. New blood vessels play a key role in tumour growth by supplying oxygen and nutrients to the tumour cells. Angiogenesis is a progressive physiological process during which specific mechanisms are induced to overcome the natural angiostatic state (in most adult tissues) of the vasculature leading to the angiogenic switch [5,6]. Some proteases such as matrix metalloproteinase 9 (MMP-9) are known to induce this switch [7]. One of the most potent mediator of angiogenesis is vascular endothelial growth factor (VEGF); a highly specific mitogen for endothelial cells [8]. VEGF system is composed of five isoforms created by alternative splicing of the *VEGF* mRNA. The human VEGF proteins consist of 121, 145, 165, 189 and 206 amino acids (VEGF_121_, VEGF_145_, VEGF_165_, VEGF_189_, and VEGF_206_). All isoforms contain exons 1–5 and 8 and differ only by various combinations of either no additional exon (VEGF_121_), or addition of exon 7 (VEGF_165_) or exon 6 and exon 7 (VEGF_189_) [9-11]. Augmented endogenous VEGF expression has been shown in various cancers and has been associated with poor prognosis and metastasis in oral squamous cell carcinoma [12]. Another important mediator of angiogenesis is angiopoietin-1 (ANG-1). During the process of angiogenesis VEGF and angiopoietins function in concert, with VEGF acting early during vessel formation, and ANG-1 acting later during vessel remodelling, maturation and stabilization [13].

In addition to well known role of controlling leukocyte trafficking in homeostasis and inflammation[14], the chemokines system also functions in tissue regulation outside the hematopoietic compartment and is involved in angiogenesis, tumour development, growth and metastasis [15,16]. Chemokine-mediated regulation of angiogenesis involves pro-angiogenic chemokines CXCL8/IL8 [17,18] interacting with CXCR2 receptor, and anti-angiogenic chemokines CXCL10/IP10 [19,20] interacting with CXCR3 receptor. Chemokines also regulate angiogenesis in a receptor-independent manner by interfering with angiogenic function of VEGF and basic fibroblast growth factor (bFGF) [21,22]. IL-8 is a very potent angiogenic chemokine and is known to play an important role in cancer progression and metastasis [17,23,24]. IL-8 has been shown to be responsible for most of the angiogenic activity induced by human oral squamous carcinoma cells [25,26].

Previously, we have shown that multifunctional proteoglycan decorin is aberrantly expressed and localized in the nucleus bound to nuclear epidermal growth factor receptor (EGFR) in human dysplastic oral keratinocytes (DOK) and malignant squamous carcinoma cells (SCC-25) [27,28]. In an effort to examine the role of this nuclear localized decorin, we stably knocked down nuclear decorin in this oral cancer cell line model. Post-transcriptional nuclear decorin knock down resulted in significantly reduced IL-8 and IL8-dependent migration and invasion in these dysplastic and malignant oral epithelia [28]. Since IL-8 is also a very potent proangiogenic chemokine, in this current study we examined the effect of nuclear decorin knockdown on angiogenesis and angiogenesis related pathways.

## Methods

### Cell lines and culture conditions

Oral epithelial origin, premalignant- Dysplastic Oral Keratinocytes (DOK) and malignant- Squamous Carcinoma Cell (SCC-25) lines were routinely maintained in DMEM/F12 (Hyclone, Logan, Utah) supplemented with 10% Foetal Calf Serum for use as *in vitro* model in our studies, as described previously [29,30]. Silencing of decorin gene expression was achieved using short hairpin RNA (shRNA) technology as explained earlier [28]. Briefly, oligonucleotides targeting decorin transcript variants A1 and A2 and scrambled sequence control were custom synthesized, annealed, and cloned into shRNA expression vector pGeneClip Puro^TM^ (Promega) by Super Array Bioscience Corporation (Frederick, MD). BLAST queries were performed to ensure that the sequences have no significant homology with any other human genes. Transformation grade shRNAi plasmids were transfected into DOK and SCC-25 cells using Effectene™ transfection reagent following manufacturer’s protocol (Qiagen, Valencia, CA). The stable transfectants were selected for puromycin (Calbiochem, San Diego, CA) resistance at 2.5 μg/ml optimal concentration. Pools of stable transfectants (maintained at 1 μg/ml of puromycin) were used in all experiments to avoid clone-specific differences. Decorin knock down was confirmed at transcript and protein level by quantitative real-time reverse transcription-PCR and Western blotting, respectively. Pooled decorin-shRNA transfected DOK or SCC-25 clones showed more than 80% reduction in decorin mRNA expression and almost complete abrogation of decorin protein expression in nuclear lysates and /or in whole cell lysates when compared to control-shRNA transfected clones or no transfection wild type DOK [28]. Herein, untransfected DOK and SCC-25 cells will be referred to as wild type (WT), scrambled shRNA stable transfectants as control (or Ctrl-shRNA in figures), and decorin shRNA stable transfectants as decorin silenced (or DCN-shRNA in figures). WT, control, or decorin silenced DOK and SCC-25 cells were cultured (5 x 10^5^ cells/ well) in complete medium for 24 h, conditioned medium (CM) was harvested and was concentrated using a Centriplus™ centrifugal concentrator (Millipore, Bedford, Massachusetts).

Human umbilical vein endothelial cells (HUVECs) (ATCC, Manassas, VA) were maintained in the endothelial cell growth medium EGM-2 Bullet Kit ® (Lonza, Walkersville, Maryland). Culture medium was changed every 2–3 days, and cells were passed when 90% confluence. Experiments were performed using cells between passages 2 and 5. HUVECs were incubated in DMEM/F12 supplemented with 10% FBS for 24 h before use in tube formation angiogenesis assay.

### Multiplex PCR

RNA was extracted from DOK and SCC-25 cells using RNeasy Plus mini kit (Qiagen, Valencia, CA). cDNA was synthesized from 2.5 μg of RNA using SuperScript™ III Reverse Transcriptase (Invitrogen, San Diego, CA). The expression levels of vascular endothelial growth factors and its receptors (VEGF 165, VEGF 121, VEGF 189, FLT1 and FLK1, Angiopoietin-1 & 2, and TIE-2), cytokines (IL-8, IL-14, IL-10, IL-5, and TGFβ) and chemokines receptors (CXCR1&2,CXCR4,) were measured in decorin silenced, control, and WT cells using multiplex PCR (MPCR) Kits for human VEGF and its receptors set-3, human TH1/TH2 cytokines set-4 and human chemokines receptors CXCR set-1(MaximBiotech, San Francisco, CA) respectively.The PCR primers in theses kits have similar Tm and no obvious 3'-end overlap to enhance multiple amplification in a single tube. The expression of the housekeeping gene, GAPDH, was also measured in each reaction. MPCR was carried out according to the manufacturer’s instructions. Briefly, 1X MPCR buffer, 2.5 units of *Taq* DNA polymerase, and cDNA template were mixed in a 25 ul reaction and subjected to 35 cycles of PCR, with denaturing, annealing, and extension temperatures at 96, 60, and 70°C, respectively. Following MPCR, the DNA amplicons were fractionated electrophoretically in 2% agarose gel containing 0.5 ug/mL ethidium bromide.

### Western blot analysis

Cells were rinsed with ice-cold PBS and were lysed in a buffer containing 20 mM Tris, pH 7.6, 0.1% SDS, 1% Triton-X, 1% deoxycholate, 100 μg/ml PMSF, and protease inhibitor cocktail (Sigma-Aldrich, St. Louis, MO). Lysates were centrifuged at 20,000 x *g* for 20 min at 4°C. Protein concentration was determined by Bis-Cinchonic Acid (BCA) protein assay (Pierce, Rockford, IL) and subjected to 10% SDS-PAGE analysis, followed by transfer to polyvinylidene difluoride membrane (Bio-Rad, Hercules, CA). The membranes were immunoprobed with 1:1000 dilution of sheep polyclonal antibody to human MMP9 ((Abcam, Cambridge, MA) or 1:1000 dilution of anti-human β-tubulin polyclonal antibody. Western blots were developed with appropriate horseradish peroxidase conjugated secondary antibodies (Bio-Rad) and ECL Plus chemiluminescence system (Amersham, Arlington Heights, IL) and exposed to auto radiographic films.

### *In vitro* angiogenesis assay

In Vitro angiogenesis was assessed using the endothelial tube formation assay (Cell Biolabs, San Diego, CA) which is based on the ability of endothelial cells to form three dimensional capillary-like tubular structures when cultured on a gel of basement membrane extract. Manufacturer’s instructions were followed to perform the assay. Briefly ECM gel prepared from Engelbreth–Holm–Swarm (EHS) tumour cells was thawed overnight at 4°C and mixed to homogeneity using cooled pipette tips. Wells of pre-chilled 48 well cell culture plate were coated with a thin layer of ECM gel (100 ul /well) and left to polymerize at 37°C for 60 min. HUVECs (3X10^4^) in 150 ul medium were added to each well along with 150 ul of either WT, control or decorin silenced conditioned medium on solidified ECM gel. The plate was incubated at 37°C for 18 h, and the endothelial tubes were observed using a light microscope. Three microscopic fields were selected at random and photographed. Tube formation ability was quantified by counting the total number of cell clusters and branching under three X4 fields per well. The results were expressed as mean of branching compared with the control groups. Each experiment was performed at least twice.

### Statistical analysis

Student’s paired *t* test was used to determine the statistical significance of the data. Statistical analysis was performed on Graph Pad Prism Software. Significance was evaluated at *p* values:

* *p* <;0.05, ** *p* <;0.01, *** *p* <;0.001.

## Results

### Effect of nuclear decorin silencing on cytokines/Chemokines expression in dysplastic and malignant oral epithelia

**Figure 1:**
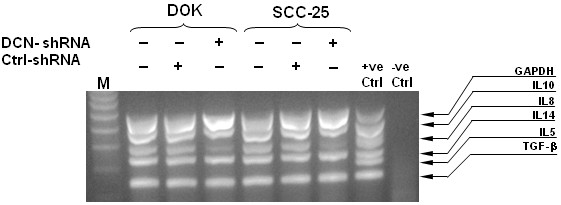
**Effect of nuclear decorin silencing on cytokines/chemokines expression in decorin silenced DOK and SCC-25 cells.** DOK and SCC-25 cells were stably transfected with decorin-shRNA (DCN-shRNA), or scrambled sequence-shRNA (Ctrl-shRNA) or no transfection control (WT). RNA was extracted and cDNA was subjected to multiplex RT-PCR to detect multiple cytokine transcripts. MPCR products were fractionated electrophoretically on a 2% agarose gel containing 0.5 mg/ml ethidium bromide, visualized under UV light and photographed. The results shown are representative of three independent experiments.

To evaluate the effect of aberrantly expressed nuclear decorin on the expression level of TGF-β, IL-10, and IL-8 simultaneously, DOK and SCC-25 WT, decorin silenced and control cells were allowed to grow in culture for 24 h, RNA was extracted and reverse transcribed to cDNA and subjected to multiplex PCR. Compared with WT or control-shRNA cells, decorin silenced DOK and SCC-25 cells did not show any change in expression level of either IL-10 or TGF-β. However, as we have previously shown [28] IL-8 expression was significantly reduced in decorin silenced DOK or SCC-25 cells (Figure 1).

### Nuclear decorin silencing down regulates IL-8 receptors in DOK and SCC-25 cells

**Figure 2:**
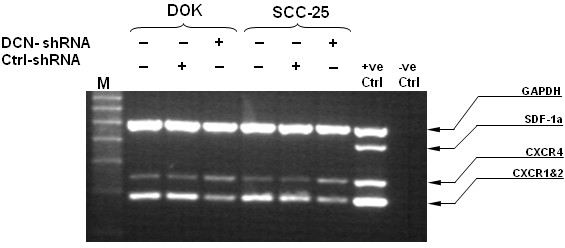
**Nuclear decorin silencing affects IL-8 receptor expression in decorin silenced DOK and SCC-25 cells.** RNA was extracted from WT, control and decorin silenced DOK and SCC-25 cells and cDNA was subjected to multiplex RT-PCR to detect CXCR1&2 expressions along with other receptors. MPCR products were fractionated electrophoretically on a 2% agarose gel containing 0.5 mg/ml ethidium bromide, visualized under UV light and photographed. The results shown are representative of three independent experiments.

We determined the expression of IL-8 receptor (CXCR1&2) along with other chemokines stromal cell-derived factor (SDF-1) and their receptors (CXCR4) in a multiplex PCR system. As shown in Figure 2, CXCR1&2 expression was found to be slightly lower in decorin silenced dysplastic or malignant epithelia in comparison to respective wild type and control cells. The expression of CXCR4 was relatively unaffected and its cognate ligand SDF-1/CXCL12 was not expressed in these cancerous and dysplastic oral epithelial cells.

### Reduction of MMP9 protein expression in nuclear decorin silenced DOK and SCC25 cells

**Figure 3:**
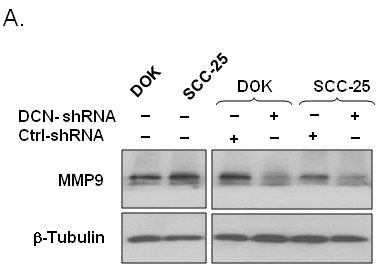
**MMP9 expression down regulation in decorin silenced DOK and SCC25 cells.** Cell lysates from decorin silenced and control dysplastic and malignant oral epithelia were collected as described in materials and methods and subjected to SDS-PAGE followed by immunoblotting using anti-MMP9 and anti-β-tubulin antibodies. Data shown from one representative experiment of three.

Matrix metalloproteinase 9 is not only involved in cancer cell migration and invasion but also triggers angiogenic switch [7] and hence plays a key role in tumour progression. Therefore, we sought to determine if nuclear decorin silencing has an effect on MMP9 expression in these dysplastic and malignant oral epithelial cells. Interestingly, MMP9 protein expression was significantly reduced in nuclear decorin-silenced DOK or SCC-25 cells as compared to control and WT cells when measured by western blotting (Figure 3).

### Effect of nuclear decorin silencing on VEGF and angiopoietin-1 expression in decorin silenced DOK and SCC25 cells

**Figure 4:**
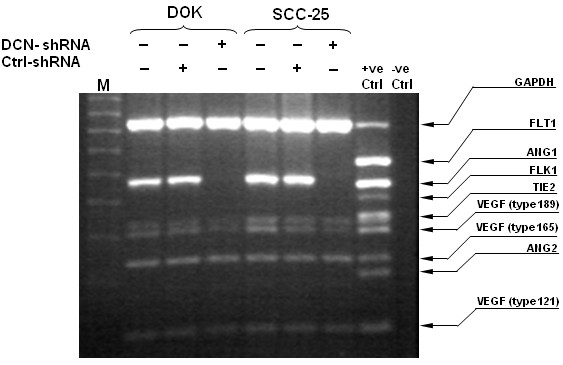
**VEGF and ANG-1 expression modulation in decorin silenced DOK and SCC25 cells.** RNA was extracted from WT, control and decorin silenced DOK and SCC-25 cells and cDNA was subjected to multiplex RT-PCR to detect the expression of angiogenesis related mediators. MPCR products were fractionated electrophoretically on a 2% agarose gel containing 0.5 mg/ml ethidium bromide, visualized under UV light and photographed. The results shown are representative of three independent experiments.

The process of angiogenesis is tightly regulated and depends on a dynamic balance between several mediators; vascular endothelial growth factors and angiopoietins are two such mediators. We determined the expression of these and other angiogenesis related mediators in nuclear decorin silenced dysplastic and malignant oral epithelial cells via multiplex PCR assays. As shown in Figure 4, the expression of ANG-1 was completely abrogated in decorin silenced DOK or SCC-25 cells, where as VEGF_189_ expression was significantly down regulated. However other VEGF splice variants VEGF_121_ and VEGF_165_ remained unaffected (Figure 4). As expected, VEGF receptors FLT1 and FLK1 were not expressed by these oral epithelial cells, and only a faint TIE2 (ANG-1 receptor) band was detected in both DOK and SCC25 irrespective of nuclear decorin silencing. ANG-2 was not expressed across the cell lines tested.

### Decorin silencing attenuates the angiogenic potential of dysplastic and malignant oral epithelial cells

**Figure 5:**
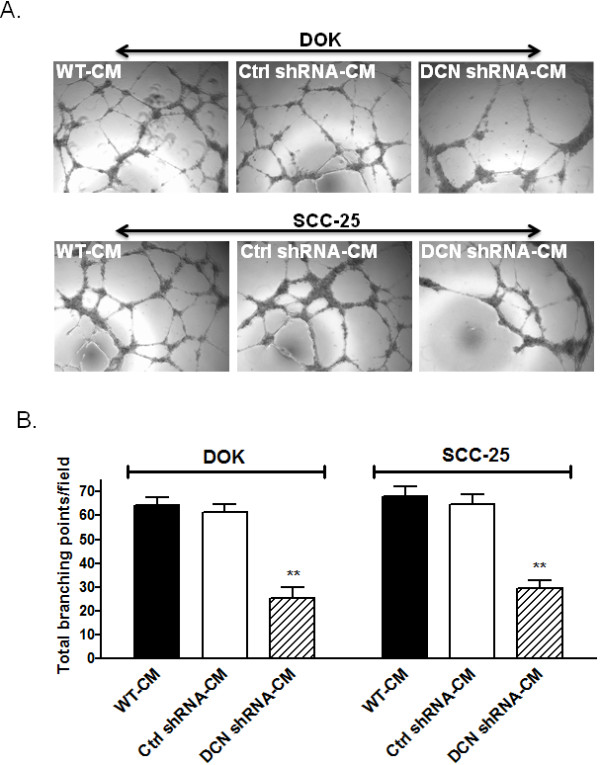
**Overall influence of nuclear decorin silencing on angiogenic capacity of DOK and SCC-25 cell derived factors; as assessed on human endothelial cells*in vitro.****A*, HUVECs (3X10^4^) were plated on ECM coated wells along with 150 ul of either WT, control or decorin silenced conditioned medium (CM). Endothelial tube formation was assessed after incubating at 37°C for 18 h, using a light microscope. Three microscopic fields were selected at random and photographed at 200x magnification. *B*, Tube formation ability was quantified by counting the total number of cell clusters and branching under three fields per well. The results are expressed as mean of branching compared with the control groups. Each experiment was performed at least twice. Numbers represent mean ± SD of three individual experiments. ** *p* <;0.01, *** *p* <;0.001 compared to respective controls.

Since nuclear decorin silencing resulted in down regulation of a variety of proangiogenic mediators, we examined whether decorin silencing has any effect on angiogenesis of oral squamous cell carcinoma. An *in vitro* tube formation assay using HUVECs was performed to mimic *in vivo* oral squamous cell carcinoma associated angiogenesis. We observed a significant suppression of vessel-like structures, consisting of elongations and alignment of the cells when HUVECS were incubated with CM from either decorin-silenced DOK or SCC-25 cells compared to respective WT or control cells (Figure 5).

## Discussion

Tumours require neovascularisation / angiogenesis to progress into clinically significant dimensions, and to metastasize [31] . They need a microvasculature to deliver micronutrients and oxygen to the growing tumour mass. Studies have indicated that an increased angiogenesis (microvascular density) correlates with poor outcomes in several human tumours [32]. Mechanistically, tumour angiogenesis involves proliferation and migration of vascular endothelial cells and their organization into tumour associated blood vessels. Several mediators of angiogenesis have been identified including VEGF, FGF, and IL8. Under normal conditions, a steady equilibrium is vigilantly maintained between proangiogenic and antiangiogenic mediators. Tumour cells acquire the ability to sustain angiogenesis by aberrant production of angiogenic or antiangiogenic factors during transition to the malignant phenotype [33].

IL-8 is a potent angiogenic factor that plays an important role in cancer progression and metastasis [17,23,24]. Importantly, it has been demonstrated that IL-8 is responsible for most of the angiogenic activity induced by human oral squamous carcinoma cells [25,26]. IL-8 (CXCL-8) is a member of the CXC chemokines family and IL-8 receptors CXCR1 and CXCR2 are expressed on various normal and cancerous cells including head and neck squamous cell carcinoma [34-36]. The mechanism(s) regulating IL-8-mediated angiogenesis, though not fully understood but is quite elaborated. Besides tumor cells, IL-8 and its receptors CXCR1 and CXCR2 have been observed on endothelial cells [24,37] and have been shown to play a role in endothelial cell proliferation [17]. A more direct role of IL-8 in angiogenesis by enhancing proliferation and survival and inhibiting apoptosis of CXCR1- and CXCR2-expressing endothelial cells have been described. Furthermore, treatment of endothelial cells with IL-8 significantly enhanced production of MMP9 and capillary tube organization [38]. We have previously shown that nuclear decorin interacts with nuclear localized EGFR in dysplastic and malignant oral epithelial cells. We have also shown that nuclear decorin knockdown significantly reduces IL-8 production in these cells. Interestingly, EGFR (trans) activation has been shown to mediate IL-8 production in epithelial cells [39,40].

As mentioned above, angiogenesis is a complex multistage process. Two important steps are endothelial cell proliferation and migration by degradation of the extracellular matrix by matrix metalloproteinase [41] and capillary tube formation mediated by various angiogenic factors such as vascular endothelial growth factor, basic fibroblast growth factor, and IL-8 [42]. Recent reports suggest that in addition to cell proliferation and migration, endothelial cell survival and death are also important components for tumor angiogenesis which is in part mediated by vascular endothelial growth factors by inducing expression of anti apoptotic protein Bcl-2 [43]. A cell cycle-regulated apoptosis inhibitor, survivin and the cell death-related gene family, Bcl-2 are associated with VEGF-induced angiogenesis [44].

Angiopoietin 1 (ANG-1) promotes angiogenesis by enhancing migration and proliferation of endothelial cells [45]. More recently, it has been shown that this positive effect of ANG-1 on endothelial cell migration and proliferation is mediated, in part, by the production of IL-8, which by acting in an autocrine fashion suppresses apoptosis and facilitates endothelial cell proliferation and migration [46].

Our current study indicates that a myriad of proteins get affected upon nuclear decorin silencing in dysplastic and malignant oral epithelial cells, including IL-8, IL-8 receptors, MMP9, VEGF, and ANG-1. Interestingly many of these proteins are linked to IL-8(in one way or another) which is a very potent angiogenic factor especially in oral squamous cell carcinoma [25]. As we have indicated before [28], nuclear decorin perhaps effects IL-8 production through its direct or indirect interactions with other normal or aberrant nuclear proteins such as nuclear epidermal growth factor receptors in dysplastic and malignant oral epithelial cells. Our recently published and current results also indicate that IL-8 activity in tumour and tumour microenvironment contributes to oral cancer progression through its functions in the regulation of angiogenesis, tumour cell invasion and metastasis.

## Conclusions

We have used *in vitro* stable gene silencing in human dysplastic and malignant oral epithelial cells to study the role of nuclear localized decorin in oral cancer angiogenesis. We demonstrate that nuclear localized decorin knockdown significantly inhibits major proangiogenic mediators such as IL-8, MMP9, ANG-1 and VEGF. The decreased expression of these angiogenesis mediators correlated with decreased vascularization capacity *in vitro*. These findings suggest that nuclear decorin silencing may suppress the angiogenesis of oral squamous cell carcinoma by inhibiting the expression of certain proangiogenic mediators, in particular IL-8.

## Competing interests

No financial or non-financial competing interests exist.

## Authors’ contributions

ND contributed to study concept and designed, performed, and analyzed the experiments, interpreted data and wrote the manuscript. AGB conceptualized the study and designed decorin targeting oligonucleotides, analyzed results and interpreted data, corrected and approved the final manuscript and directed the research program. All authors reviewed and approved the final manuscript.
